# Loot

**Published:** 1869-11

**Authors:** 


### Loot.

### *On Certain Influences of Schools Injurious to Health.*

BY PROF RUD. VIRCHOW.* *Virchow's Archiv., XLVI*, p. 447, May, 1869.

THE injurious influences of schools upon the health of the pupils have frequently, especially since the end of the past century, attracted the attention of physicians and teachers, now on this, now on that point, yet usually without being investigated more exactly, in a truly scientific manner. . . . . Only very few attempts were made, by means of systematic researches, to gain a basis of real facts for forming opinions, and it must be acknowledged a very great advance that a beginning has been made at present in certain directions to procure statistics of what may be called the school diseases. Only extensive, scientifically certain, *comparative* statistics, will enable us to judge, with full confidence, what affections or diseases are produced by the schools,

* This report owes its origin to an investigation by request of the Prussian Government. We consider it of considerable value, presenting a clear and convenient view of this important hygienic question, and therefore give an English version of as much of it as our space will allow.—ED.

and what means should be adopted to prevent them. Where this basis is wanting, there are still certain general scientific rules, which are applicable to schools as well as to other institutions of society, but it can not be denied that, in applying them, important conditions may very easily be overlooked or wrongly estimated.

The following report will endeavor to keep the two groups as distinct as possible, and carefully separate those affections well established in fact from those merely guessed at. In first line, as regards reliability of actual demonstration, we have:

1. *Affections of the Eye, especially Near-sightedness.* The first (though still inaccurate, and in no wise methodical) endeavors to establish, by statistics, the supposed influence of schools upon the development of near-sightedness, were made in the beginning of this century, by the Englishman Ware. Since then, similar researches have been made in different parts of the continent, particularly in Germany, partly official, partly by private persons, but almost never logically and systematically. Only the investigations of Dr. Hermann Cohn,* of Breslau, both as regards the number of persons examined, and as regards the method and accuracy of observation, have assumed a shape corresponding to the demands of modern science, and may be considered as exceedingly valuable, nay, in a certain sense, as decisive. . . . .

The total result was found to be that, among the 10,060 scholars, vision was not normal in 17.1 per cent., but that the latter figure was very unequally distributed, namely:

| | |
|---|---|
| In the village schools, - - - - - | 5.2 per cent. |
| " city primary schools, - - - - - | 14.7 " |
| " middle schools, - - - - - | 19.2 " |
| " girls' high schools, - - - - - | 21.9 " |
| " "Real" schools, - - - - - | 24.1 " |
| " gymnasia [high schools], - - - | 31.7 " |

Among the 410 students examined there were found even 68 p. c. with abnormal vision (ametropia).

Now, leaving out of present consideration hyperopia, astigmatism, and the actual *diseases* of the eyes, as of secondary importance, and considering only near-sightedness proper (myopia), still we meet with the distressing result, that in all nearly 10 p. c. of the children are near-sighted, viz.:

| | | |
|---|---|---|
| In village schools, - - - | 1.4 p. c. | |
| " city primary schools, - - - | 6.7 " | city schools 11.4 p. c. |
| " girls' schools, - - - | 7.7 " | |
| " middle schools, - - - - | 10.3 " | |
| " "Real" schools, - - - | 19.7 " | |
| " gymnasia, - - - - | 26.2 " | |
| And among students, - - - | 60.0 " | |

* Some account of these investigations was presented to the readers of this [St. Louis] journal in the January number of this year, p. 73.

The regular increase here presented in the aggregate is repeated in a remarkable manner in taking separate account of each school according to classes:

| | VI.* | V. | IV. | III. | II. | I. |
|---|---|---|---|---|---|---|
| Primary schools, - - | — | — | 2.9 | 4.1 | 9 8 | 9.8 |
| Gymnasia, - - - | 12.5 | 18.2 | 23.7 | 31.0 | 41 3 | 55.8 |

The unfavorable opinion of Dr. Cohn is, therefore, unfortunately, incontrovertible, the less so since he shows, by extensive tabular evidence, that not alone does the number of the near-sighted increase from class to class, but the degree of myopia likewise rises. . . . . The myopia in these schools, therefore, is on the whole *progressive;* it pursues that dangerous path which gradually leads to actual asthenopia.

Dr. Cohn justly disavows the opinion that the enormous frequency of myopia among school children should be ascribed solely and exclusively to the school. Outside of the school, even under the parental roof, many unfavorable conditions are evidently operating. . . . . Nevertheless, it may be affirmed, with full assurance, that persons of the age of scholars in the first class of gymnasia do not average 55 to 56 p. c. of near-sighted, nor are there 60 p. c. among persons of the age of students. And though we may concede that insufficient illumination, fine print and fine handwriting, the habit of bending forward, etc., operate injuriously in domestic occupations, also, it must yet be acknowledged that some of these disadvantages at home arise from habits of the school, or at least that the school does not sufficiently oppose the acquiring of these habits, but rather directly favors some of them.

Beside the question of illumination and light of the school-room, Dr. Cohn has principally examined that of the furniture, *i. e.*, desk and seat, and believes himself justified in condemning the present arrangement as positively injurious. This arrangement, he says, compels the pupils to look at print in close proximity, and with their heads bent forward. This requires, on the one hand, a greater activity of the muscles of accommodation in the eye, causing in its part an increase of hydrostatic pressure in the posterior part of the eye-ball and elongation of its axis; and, on the other hand, the bending forward of the head causes obstruction to the flow of blood from the eye, congestion of the eye, and hence, also, increased pressure in the fundus of the eye. Both circumstances together are the cause of the near-sightedness. . . . .

2. *Determination of Blood to the Head.* In the previous section the fact has already been spoken of, that the inclined position of the head causes congestion. . . . . Other conditions

* VI, or IV, respectively, being the lowest, I the highest class. — Ed.

operate in the same direction. In bending the head forward, the trunk naturally is also inclined forward, and the more so, the lower the desk. From this results a certain compression of the abdomen, with consequent impaired activity of the diaphragm, the most powerful muscle of respiration. But incomplete inspiration opposes the return of blood from the veins of the neck into the chest.

Added to this is the circumstance that during close attention respiration is incompletely performed, the more so, the less the demand for respiration is directly excited by one's own speaking. . . . .

All these circumstances favor what is called *passive* or mechanical congestion, by impeding the return of venous blood. But there is, in school, a very efficient cause also for so-called *active* congestions to the head, *i. e.*, increased flow of arterial blood, and that is the active labor of the brain. . . . .

Among the various affections arising from these partly passive, partly active, congestions, the following three have of late given occasion for statistical researches:

(*a.*) *Headache.* Guillaume (*Hygiène scolaire. Genève*, 1864), who designates it plainly as "*céphalalgie scolaire*," found, among 731 pupils of the Collége municipal in Neufchatel, 296 suffering frequently from headache,—over 40 p. c. Girls were more often afflicted with it than boys, 51 p. c. of the former, to 28 p. c. of the latter. . . . . Becker (*Luft und Bewegung zur Gesundheitspflege in den Schulen*, Frankfurt a. M., 1867) examined 3,564 pupils; . . . 974 of them, or 27.3 p. c. suffered more or less from headache. . . . . In the first class of the gymnasium 80.8 p. c. complained of it. Becker concludes from his figures—what is not quite accurate—that the number is smallest in the first school years, and increases with longer attendance, greater number of hours of attendance, and the amount of mental exertion required. As an auxiliary cause he mentions too small rooms.

It is necessary to mention another circumstance for consideration. Deville and Troost found that certain gases, especially carbonic oxide, are capable of passing through red-hot iron, a circumstance not rarely confirmed in school-rooms heated by iron stoves. Headache, dizziness, trembling and similar accidents are the consequences of a slight operation of this so poisonous gas. Dr. Oidtman does not hesitate to assume chronic poisoning by carbonic oxide as comparatively frequent in his part of the country, which is rich in iron stoves.

(*b.*) *Hæmorrhage from the Nose.* Guillaume found it frequent in 21 p. c.; more frequent in boys (22 p. c.) than in girls (20 p. c.) . . . Becker found only 11.3 p. c. subject to epistaxis; accurate details are not given, but he states that the hæmorrhages are most frequent in the higher classes of the gymnasium, in the girls' high school, and one private school, — in those schools, as he says, the pupils of which sit longest in school, and move about least in the open air.

(*c.*) *Goitre.* [Guillaume calls it *goître scolaire*, the scholars call it "*gros cou.*" He found it in 56 p. c.! — 58 p. c. among boys, 64 p. c. in girls. It frequently disappears during vacations, and becomes permanent only at a later date, but is sometimes found in girls of eight years after one year's attendance. Thus far, the data of Guillaume stand alone, and it is doubtful if they are entitled to general acceptance, though it is true that the female sex and youth predispose to goitre, and that dilatation of the cervical vessels engender a disposition to this affection.]

Headache and bleeding from the nose, however, are affections sufficiently well known to physicians, and many parents, as not rarely accompanying attendance at school. The observations before us do not indeed suffice for drawing absolutely certain conclusions, . . . nevertheless, we can but acknowledge, even now, that the school favors, and perhaps often causes, these conditions, and that their frequent occurrence should be a subject of serious consideration.

At this point, the question naturally suggests itself as to the influence of congestive states, such as have been spoken of above, in relation to the mental faculties of the pupils. In fact, it can not be doubted that such conditions are often combined with confusion, incapacity for thought and mental labor, and that, when they become habitual, they may engender dangerous predispositions of the brain. . . . .

3. *Curvatures of the Spine.* Not a few among the physicians who have paid special attention to the school question, and a great number of orthopædists, maintain the opinion that the school bears a large share of the blame in the production of deviations of the spinal column. Especially the lateral curvature, so-called scoliosis, and chiefly the form known as "habitual scoliosis," is here accused. Fahrner says: "Since almost 90 p. c. of these curvatures originate during school years, and the curvature corresponds exactly to the posture in writing, we certainly are justified in accusing the school as the main cause." Guillaume illustrates the comparison of the common form of scoliosis to the writing posture by a drawing, undoubtedly correct in itself, and adds that he found, among 731 pupils, 218 (*i. e.*, almost 30 p. c.) showing a deviation of the spine.

The experiences of orthopædists unanimously agree in that the majority of scolioses originates during the period of obligatory attendance at school. . . . . It can be asserted with safety, that the common scoliosis is a developmental disease of the age of obligatory attendance. It is less certain if the school, as such, be the chief cause of this disease. On the one hand, comparisons are wanting with such countries and ages where school attendance is not obligatory. The testimony of the Primary School Committee of New York, which Guillaume adduces, has a certain value, but it is not decisive. On the other hand, comparison of many schools would be necessary on this point. . . . . A special consideration against the implication of the school is the great preponderance of scoliosis in the female sex. [Guillaume counts 18 p. c. among boys, and 41 p. c. among girls. This, of course, includes many slight cases. The experience of orthopædists, chiefly on severer cases, is more striking. Klopsch reckons 84–89 p. c. of all scoliotic persons to be in females, etc.] With these figures before us it can not be doubtful that the school is not the only cause of scoliosis; nay, it must be conceded that it is not even the chief cause. . . . .

From consideration of the evils spoken of, even though they are only in part to be ascribed to the school, very definite obligations of the latter may be deduced. On the one hand, the pupils, especially the female pupils, must be appropriately seated and carefully watched in regard to posture and carriage. On the other hand, they must be offered well-timed opportunity by gymnastics to give proper exercise to their limbs.

4. *Affections of the Organs of the Chest*. . . . . Selecting [from the accurate mortuary tables of Berlin] the ages of obligatory school attendance, we meet with a rapid rise of the mortality from pulmonary and laryngeal phthisis in the ages of from 10 to 15 years, which began in the preceding period of from 5 to 10 years of age, and is considerably augmented still in the later period of from 15 to 20.

| | | Pulm. Phthisis. | Consumption. |
|---|---|---|---|
| There occur, at the age of | 5—10 years, | 4.81 | 8.93 |
| " " | 10—15 " | 12.96 | 7.90 |
| " " | 15—20 " | 31.88 | 4.74 per cent. |

of the deaths, from all causes, without considering scrofula and many other nearly related categories. This result is certainly very remarkable, especially when we consider that, in persons of these ages, only typhoid fever and cholera afford mortality figures at all approaching the above.

This mortality, it is true, can not be ascribed to attendance at school alone; . . . nevertheless, the fact must not be underrated. Circumstances of moment indicate that the school contributes much towards it. The following influences, more especially, may be counted as injurious: (1) the vicious air, rendered so by the presence of many children; (2) frequent "colds," caused by the change from the hot school-room to the free and cool air outside, by draughts from windows and doors, etc., whereby inflammations of the throat and chest are produced in great number;

(3) the dust of the school-rooms; (4) the fact that the respiratory movements are impaired by the long-continued sitting.

[The author here mentions that from recent investigations we are no longer able to identify phthisis with tuberculosis, but that the former is due to a variety of processes, all of which finally produce ulcerations of the lungs.] The majority of these begin with simple catarrhal and inflammatory processes, which owe their origin to external agents, especially cold and the inhalation of irritating substances (dust, carbon, etc.) Their continuation is favored by low, respiratory movements, which effect accumulations and retention of excretory matters; furthermore, by the viscidity and perishable nature of these excretory substances, which are decomposed and inspissated, and on the character of which the nature of the inspired air has no less, perhaps even more, influence than the quality of the food; finally, by the continuation or repetition of the irritations.

This brief synopsis will suffice to show how dangerous may be a school with defective arrangements and inefficient superintendence, and how much reason there is to fear that a part of the fatal results of phthisis in the school ages may really be attributed to the school as such, and that in part even the unfavorable termination taking place after school life may be laid to the school period.

5. *Affections of the Abdominal Viscera.*

6. *Contagious Diseases.*

7. *Injuries.*

[We pass over these sections for want of space, because they are of less importance, and no accurate statistical data are adduced. The author finally enumerates the following known injurious agencies and causes of disease pertaining to schools, to which attention should be directed:]

(1.) *The air in the school-room*, the quality of which is determined by the size of the room, the number of pupils, the mode of heating, the ventilation, moisture of the floor and walls, dust (cleanliness).

(2.) *The light*, as determined by the situation of the building and room, the size of the windows and their relation to the desks, the color of the walls and surroundings, artificial light (gas, oil).

(3.) *The sitting* in the school-room, especially the relations of desk and seat, size of the seats, their arrangement, duration of sitting.

(4.) *Bodily exercise*, especially playing, gymnastics, swimming, their relations to sitting and to the purely mental labor, their arrangements and superintendence.

(5.) *Mental exertion*, its duration and variety, the individual amount, the arrangement and duration of recesses and vacations, the extent of home and school exercises, the date of commencement of obligatory attendance, etc.

(6.) The *punishments*, especially corporeal.
(7.) The *water for drinking.*
(8.) The *privies.*
(9.) The *means* (implements) *of instruction*, especially the choice of school books (size of type), and objects of illustration.—*St. Louis Med. and Surg. Journal.*

Ozæna is said to have been cured (three cases) by irrigations of the part with solution of permanganate of potash, 5 parts to 100 of water.

Sycosis is successfully treated by Mr. Stewart with a saturated solution of nitrate of potash, applied three or four times a day. If it produces too much pain it may be diluted.

Phagedenic chancres were arrested in two cases, by Prof. Schwanda, by passing through them the constant electrical current.

Diphtheria was treated by the late Dr. Magruder, of Washington, as it is claimed, with most marked success, on the following plan:

The throat was rubbed frequently, externally, as soon as the swelling and soreness commenced, with spirits of turpentine. Internally, this:

| | | |
|---|---|---|
| ℞. | Potassæ chlorat., - - - - - | ʒ j; |
| | Tinct. guaiaci comp., - - - - - | ʒ ij; |
| | Tinct. cinchonæ, - - - - - | ʒ ij; |
| | Mellis, - - - - - - - | ℥ ss; |
| | Aq., - - - - - - - | ℥ iijss; |

M.—S. A tablespoonful every three hours. Twenty drops of the muriated tinct. of iron, an hour and a half after each dose.

It is said that 82 out of 85 cases were successfully treated.

Toothache finds another remedy in a paste made of nitric ether and sulphate of alumina, and applied to the cavity. The most violent odontalgia is promptly relieved, with no chance of injury, and after several applications the aching part is thoroughly anæsthetized.

Common catarrh is treated by Dr. Scudder, thus:

| | | |
|---|---|---|
| ℞. | Tinct. verat., - - - - - | ʒ ss; |
| | Tinct. gelsemini, - - - - - | ʒ j; |
| | Aq., - - - - - - - | ℥ iv; |

M.—S. A teaspoonful every one or two hours.

The same gentleman treats Neuralgia in feeble persons, or where there is a feeble circulation, with the following, used alternately:

℞. Tinct. aconit. rad., - - - - gtt x;

Tinct. belladonnæ, - - - - - gtt xx;

Aq., - - - - - - - ℥ iv;

M.

℞. Tinct. nucis vomic., - - - - ʒ j;

Aq., - - - - - - - ℥ iv;

M.—S. A teaspoonful every one or two hours.

As prophylactic in Croup, he commends a liniment:

℞. Ol. stillingiæ, - - - - -

Ol. cajeput., - - - - - aa ʒ ij;

Ol. lobeliæ, - - - - - - ʒ j;

Alcohol, 76° - - - - - - ℥ ij;

M.—Direct the mother to rub it on the throat when symptoms are manifested; and if necessary, give a drop on a lump of sugar.

***Pertussis.*** (Valentine Mott).

℞. Acid hydrocyan., - - - - - gtt. vj;
Ext. belladonnæ, - - - - - gr. ij;
Tr. opi., camph., - - - - - -
Aq. pur., - - - - - - - aa ℥ iij;
Syrup. tolutani, - - - - - ℥ j;

M.—S. A teaspoonful four times a day.

***Sore Nipples.*** (J. G. Wilson).

℞. Plumbi nitratis, - - - - - gr. x;
Glycerin., - - - - - - ℥ j;

M.—Ft. solut.

S.—Apply freely to the excoriated part.

***Trismus Nascentium.***

Dr. T. L. Papin (*Humboldt Med. Archives*), treated a case thus:

℞. Chloroform, - - - - - - *m* xij;

Sp. æth. sulph., - - . - - *m* xv;

Sodæ bicarb., - - - - - - gr. xij;

Syrup. zingiber, - - - - - -

Aq. flor. aurant., - - - - - aa ʒ iv;

M.—S. A teaspoonful p. r. n. to allay spasmodic action and secure rest and quiet.

The paroxysms after a while recurring, he resorted to the local application of chloroform to the spine, a small strip of cotton cloth wetted and applied to the entire length. This application, alternated with emollients when vesication occurred, occasional inhalations and mild purgatives was continued *fifteen* days, when the cure was complete.

Dr. Boisliniere cured a similar case, several years since, administering the chloroform by enema.

Dr. Lehlbach admires the use of carbolic acid, exceedingly. In Impetigo, he uses this ointment:

| | | |
|---|---|---|
| ℞. | Acid. carbol. crystal., - - - - - | ʒj; |
| | Sodæ sulphit., - - - - - - | ℥ss; |
| | Glycerin, - - - . - - | ℥jss; |
| | Cerat. simp., - - - - - - | ℥ij; |

M.—S. Apply three times a day.

In Scabies, he uses:

| | | |
|---|---|---|
| ℞. | Ac. carbol. cryst., - - - - | ʒjss; |
| | Flor. sulphur., - - - - - | ℥ss; |
| | Sodæ sulphit., - - - - - | ℥ss; |
| | Glycerin, - - - - - - | ℥ij; |
| | Cerat. simp, - - - - - | ℥ij; |

M.—S. Take a bath or wash all over, morning and night, and then anoint thoroughly wherever the disease extends.

The acari are generally killed in three days.

In the Diarrhœa of Children, he thinks it is particularly useful in arresting fermentation, etc. For a child from a year to eighteen months of age, he prescribes:

| | | |
|---|---|---|
| ℞. | Pepsin, - - - - - - | ʒss—ʒj; |
| | Bismuthi subnit., - - - - - | ℈ss; |
| | Pulv. opi., - - - - - - | gr. 1-5—1-2; |
| | Acid. carbol. c., - - - - - | gr. 1-8—1-2; |
| | Quiniæ sulph., - - - - - | gr. j—ij; |

M.—In chart. x divid.

S.—One every two or four hours. In some cases he substitutes bromid. potassii for the opium.

Primitive Chancres, whether soft or indurated, are treated by M. Champouillon with powdered camphor. Cicatrization takes place in ten or twelve days in ordinary cases. He thinks bubos less likely to occur. The powder is applied twice a day, taking care *not* to remove the paste formed on the surface oftener than once in two or three days.

Which reminds us that we have seen cases, where *finely pulverized loaf sugar* used in the same way, seemed to accelerate the cure remarkably.

In a case of Hæmoptysis, when local irritation was desired, Dr. Bemiss used this liniment:

℞. Hydrarg. bichlorid., - - - - . gr. ij;
Tinct. iodinii, - - - - - ʒ iv;
Glycerin., - - - - - -
Aq., - - - - - - aa ℥ ij:

M. — Moisten lint or cotton with this and apply p. r. n.

Phthisis is treated by Surgeon Sanger (*Lond. Lancet, Sept.*, 1869), with arsenic. He uses the liquor arsenicalis or solution of arsenite of soda in five minim doses, twice a day, combined with a drachm of the citrate of iron wine, or five grains of the citrate of iron and quinine. He exhibits it after eating, and orders it suspended if gastric irritation occurs. In some cases, where iron cannot be borne, he combines each dose with 15 or 20 grains of hyposulphite of soda.

A better plan is to give the Fowler's solution in regularly diminishing doses, and be guided by the influence on nutrition.

***Varicose Ulcers.***

Several cases are reported in the *Medical Gazette*, cured by Prof. Stephen Smith's method. Dr. Osborn, the reporter, finds it highly satisfactory. It is simple and safe.

"A bandage is tightly drawn around the leg, immediately below the knee, with a compress over both or either of the large veins involved. The patient is requested to throw his weight on the limb to be operated on, and when the vein becomes sufficiently distended, with a hypodermic syringe, having a sharp point, well oiled, inject into the veins below the compress, 3 or 4 minims of liquor ferri persulphatis; withdraw it immediately, placing the finger over the point of puncture, then cover it carefully with adhesive strips (icthyocoll). A fine clot will form from the compress down half an inch to an inch. All air must be forced out of the syringe before throwing in the injection. The compress may be loosened a few hours after the operation, and in a few days it may be removed entirely. The patient is enjoined to rest in a recumbent position for a few days."

This is a very easy and ordinarily safe method of treating these, frequently very troublesome sores, as well as for avoiding danger of rupture of the distended veins.

***Drowning.***

Dr. Benjamin Howard, of New York, has devised the following method for the restoration from drowning, which seems preferable, in some respects, to the plans usually resorted to, and is well worthy of having its merits tested by careful trial of it:

1. Unless in danger of freezing, never move the patient from the spot where first rescued, nor allow bystanders to screen off the fresh air, but instantly wipe clean the mouth and nostrils, rip and remove all clothing to a little below the waist, rapidly rub dry the exposed part, and give two quick smarting slaps on the stomach with your open hand.

If this does not succeed immediately, proceed according to the following rules to perform artificial breathing:

2. Turn the patient on his face, a large bundle of tightly rolled clothing being placed beneath his stomach, and press heavily over it upon the spine for half a minute.

3. Turn the patient quickly again on his back, the roll of clothing being so placed beneath it as to make the short ribs bulge prominently forward, and raise them a little higher than the level of the mouth. Let some bystander hold the tip of the tongue out of one corner of the mouth with a dry handkerchief, and hold both hands of the patient together, the arms being stretched forcibly back above the head.

4. Kneel astride the patient's hips, and with your hands resting on his stomach, spread out your fingers so that you can grasp the waist about the short ribs. Now, throw all your weight steadily forward upon your hands, while you at the same time squeeze the ribs deeply, as if you wished to force every thing in the chest upwards out of the mouth. Continue this while you can slowly count — one — two — three; then suddenly let go, with a final push, which springs you back to your first kneeling position. Remain erect upon your knees while you can count — one — two; then throw your weight forward again, as before, repeating the entire motions — at first, about four or five times a minute, increasing the rate gradually to about fifteen times a minute, and continuing with the same regularity of time and motion as is observed in the natural breathing which you are imitating.

5. Continue this treatment, though apparently unsuccessful, for two hours, until the patient begins to breathe; and for a while after this help him, by well-timed pressure, to deepen his first gasps into full, deep breaths; while the friction of the limbs, which should, if possible, have been kept up during the entire process, is now further increased.

6. After-treatment — externally: As soon as the breathing has become perfectly natural, strip the patient rapidly and completely. Enwrap him in blankets only. Put him in bed in a room comfortably warm, but with a free circulation of fresh air, and except for the administration of internal treatment, let him have perfect rest.

Internally: Give a little hot brandy and water, or other stimulant at hand, every ten or fifteen minutes for the first hour, and as often thereafter as may seem expedient.

### *Cutaneous Affection Caused by the Use of Bromide of Potassium.*

Bromide of potassium has been so largely employed of late years in the treatment of a great variety of diseases, that it is well to bear in mind that it may itself induce certain cutaneous affections, and these have been particularly noted by M. Voison. He enumerates no less than five, namely:

1. An eruption resembling acne, affecting principally the face and chest, accompanied by more or less itching, and rarely with fever. This may occur when not more than a drachm has been ingested.

2. Amongst ninety-six epileptics treated with bromide of potassium, there occurred, six times, large, elongated, rose or cherry-red swellings, half an inch or more in diameter, on the surface of which collections of pustules, also resembling acne, were developed. The swellings occurred invariably on the lower extremities, and were extremely painful. They healed very slowly, thick crusts forming, leaving indelible yellow spots, covered with scales.

3. On two occasions an exanthematous eruption, resembling erythema nodosum, appeared, though unaccompanied by any febrile symptoms, which lasted for more than six months.

4. Very troublesome furunculosis, or succession of boils with carbuncles on the neck.

5. In one instance an eczematous eruption appeared on the legs, which lasted for a year, and was accompanied by profuse secretion. In this instance, also, the greater part of the head was affected with pityriasis.

The treatment recommended by M. Voisin, as generally applicable for these cutaneous affections, consists in the administration of alkaline drinks and baths, sudorifics, local emollients, etc. — *Practitioner*, *Oct.*, 1869, *from Centralblatt.*

### *Therapeutic.*

Mr. Editor. — I would like to ask, in your journal, for the remedy *warranted* to cure a case of leucorrhœa, in a young girl, aged 15; well developed physically — menses well established — free and regular — health apparently good, but continually troubled with a discharge, staining the linens yellow; perhaps patient rather scrofulous, inclined to increased secretions from mucous surfaces. J. L. W.

We never *warrant* our patients any thing, except that we will do our best. For the case, as it stands, we would suggest Sep., and recommend a "voyage of discovery." Doctor, are there no concomitant symptoms? Look for a cause. A case somewhat similar was caused by a *spool of thread!* — *Med. Investigator.*

### *External Use of Digitalis as a Diuretic.*

Dr. Brown has succeeded in re-establishing the renal function in six cases of calculus of the kidney, when all other measures had been tried without effect, by the external use of digitalis in the form of poultices made either by throwing the fresh leaves into boiling water, or by incorporating the concentrated tincture with linseed meal. A rapid fall of the pulse follows the application. The cataplasms made with the leaves are especially to be recommended, and they should be renewed every six hours till the lowering of the pulse warns us to desist. — *Dublin Quart. Journal Med. Sci., August*, 1869, *from Rev. de Thér., Dec.*, 1868.

Which reminds us of old-time usages. Here are a couple of ancient prescriptions for the production of diuresis:

| | | |
|---|---|---|
| ℞ | Tinct. Digital., Tinct. Scillæ, - - - - | |
| | Linim. Sapon. and Opi., - - - - | aa ℥ ij; |
| | Aq. Ammon., Ol. Camph, - - - | aa ℥ ss; |
| | Tinct. Cantharid. Fort., - - - - | ʒ ij; |

M. — S. To be rubbed in freely, over the surface, three times a day.

| | | |
|---|---|---|
| ℞ | Liniment. Volatil., - - - - - | partes ij; |
| | Tinct. Cantharid., Tinct. Digital., - - | |
| | Tinct. Colchici, Tinct. Iodin., - - - | aa p. j; |

M. — S. Apply over half the body, three times a day.

### *Management of After-pains.*

Prof. T. Gaillard Thomas, M.D., in a "Lecture on the Management of Woman after Parturition," gives us the following timely hints:

The less firmly a uterus contracts, the more severe are the after-pains, for firm contraction expels these clots, and thus the cause of the painful contraction disappears. For this reason, after first labors, they do not occur. To cause firm uterine contraction, administer a full dose of ergot, just after, or during the stage, in every case. Ergot never does harm, but fulfills an indication of great importance, and guards against the liability of hæmorrhage. Should this means fail, let the opiate be given before the obstetrician leaves the house. An excellent combination, which he habitually employs for this purpose, is the following:

| | | |
|---|---|---|
| ℞ | Chloroformi (Squibbs), - - - - - | ℥ ijss; |
| | Morphiæ Sulph., - - - - - - - | gr. ijss; |
| | Syrupi, - - - - - - | ℥ ij; |

M. — A teaspoonful every hour till relieved. — *Rich. and Lou. Med. Jour.*

